# Surface Free Energy Dominates the Biological Interactions of Postprocessed Additively Manufactured Ti-6Al-4V

**DOI:** 10.1021/acsbiomaterials.2c00298

**Published:** 2022-09-21

**Authors:** Victor Manuel Villapun Puzas, Luke N. Carter, Christian Schröder, Paula E. Colavita, David A. Hoey, Mark A. Webber, Owen Addison, Duncan E. T. Shepherd, Moataz M. Attallah, Liam M. Grover, Sophie C. Cox

**Affiliations:** † School of Chemical Engineering, University of Birmingham, Edgbaston B15 2TT, U.K.; ‡ School of Chemistry, CRANN and AMBER Research Centres, Trinity College Dublin, College Green, Dublin 2 D02 PN4, Ireland; § Trinity Biomedical Sciences Institute, Trinity College, Trinity Centre for Biomedical Engineering, Dublin D02 R590, Ireland; ∥ Department of Mechanical Manufacturing and Biomedical Engineering, School of Engineering, Trinity College, Dublin D02 DK07, Ireland; ⊥ Quadram Institute Bioscience, Norwich Research Park, Colney NR4 7UQ, U.K.; # Norwich Medical School, University of East Anglia, Norwich Research Park, Colney NR4 7TJ, U.K.; ∇ Faculty of Dentistry, Oral and Craniofacial Sciences, King’s College London, London SE1 9RT, U.K.; ○ School of Engineering, University of Birmingham, Edgbaston B15 2TT, U.K.; ◆ School of Materials and Metallurgy, University of Birmingham, Edgbaston B15 2TT, U.K.

**Keywords:** additive manufacturing, medical devices, powder bed fusion, biological interactions, physicochemical characterization

## Abstract

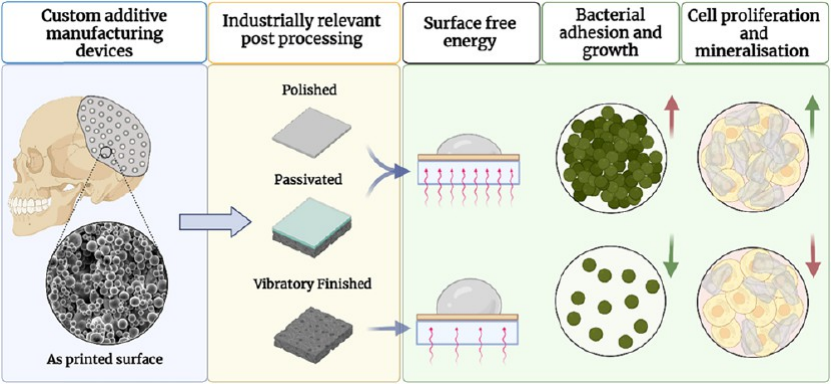

Additive manufacturing (AM) has emerged as a disruptive technique within healthcare because of its ability to provide personalized devices; however, printed metal parts still present surface and microstructural defects, which may compromise mechanical and biological interactions. This has made physical and/or chemical postprocessing techniques essential for metal AM devices, although limited fundamental knowledge is available on how alterations in physicochemical properties influence AM biological outcomes. For this purpose, herein, powder bed fusion Ti-6Al-4V samples were postprocessed with three industrially relevant techniques: polishing, passivation, and vibratory finishing. These surfaces were thoroughly characterized in terms of roughness, chemistry, wettability, surface free energy, and surface ζ-potential. A significant increase in *Staphylococcus epidermidis* colonization was observed on both polished and passivated samples, which was linked to high surface free energy donor γ^–^ values in the acid–base, γ^AB^ component. Early osteoblast attachment and proliferation (24 h) were not influenced by these properties, although increased mineralization was observed for both these samples. In contrast, osteoblast differentiation on stainless steel was driven by a combination of roughness and chemistry. Collectively, this study highlights that surface free energy is a key driver between AM surfaces and cell interactions. In particular, while low acid–base components resulted in a desired reduction in *S. epidermidis* colonization, this was followed by reduced mineralization. Thus, while surface free energy can be used as a guide to AM device development, optimization of bacterial and mammalian cell interactions should be attained through a combination of different postprocessing techniques.

## 1 Introduction

Although the complication rate in prosthetic joint replacements is low, solely in the United States, the annual costs of revision surgeries were estimated to be $1.62 billion in 2020.^1^ As the population grows older, the number of orthopedic interventions is expected to exceed 5 million worldwide by 2021.^2^ As such, there is a critical need to develop devices with minimal failure rates resulting from limited biocompatibility, aseptic loosening, and infection.^3,4^ To alleviate and prevent such outcomes, implementation of novel materials and processes has become crucial for which additive manufacturing (AM) has been the lead disruptor in orthopedic device manufacture. Their ability to produce bespoke implants^5^ and versatility to enable design modifications that may improve osseointegration, reduce stress shielding, incorporate therapeutically loaded materials, or reduce magnetic resonance imaging artifacts provide further rationale to adopt these technologies in healthcare.^6−8^ Despite the great benefits brought by AM, a limited understanding of both eukaryotic and prokaryotic cell–surface interactions poses a risk to their successful implementation.

Even though numerous materials can be applied in healthcare, the field of prosthetics has demonstrated a preference for titanium alloys manufactured through laser powder bed fusion (PBF-LB).^5^ In this technology, an energy source selectively melts powdered particles allowing for structural control and customization from the micro- (∼150 to 250 μm) to macroscales. Although this layer-by-layer approach offers a flexible manufacturing process, the localized melting and highly directional heat flow coupled with surrounding powder results in heterogeneous microstructures and poor surface finish for as-printed devices^9,10^ which can result in mechanical failure, cytotoxicity, and implant rejection.^11,12^ These defects have historically been addressed through a combination of process optimization and physicochemical treatments. Parametric analyses have showed promise on limiting surface defects; nevertheless, surface postprocessing in the form of polishing, passivation, vibratory finish, or sand-blasting is still heavily used in the AM field.^5,13^ The available literature on AM postprocessing aims to reduce poor surface finish to enhance mechanical strength and fatigue performance. In this regard, all aforementioned techniques have shown their ability to improve the rough as-build surface, *R*_a_ > 13 μm to micron and submicron values, limiting crack initiation sites.^14^ However, relatively few studies have focused on their influence on subsequent eukaryotic cell and microbial attachment of AM surfaces. Most of the available work aims to compare some of these techniques in conventional titanium alloys.^15^ This is surprising as, while polishing may solely remove partially adhered particles, etching, blasting, and vibratory finish alter the oxide layer of AM parts or may leave contaminants, endangering the biological outcomes of the device.^5^ Given the peculiarities of the AM process and surface finish, the few available studies focused on AM have been able to demonstrate the ability of microstructures obtained through postprocessing^16,17^ or part orientation^18,19^ to control biological outcomes. However, our limited knowledge of cell–surface interactions on AM devices indicates that a fundamental study is necessary.^20^

The catastrophic effect of bacterial colonization, biofilm formation, and aseptic loosening on implantable devices has led to broad interest in unraveling the mechanisms behind cell adhesion to biomaterials.^21,22^ In the case of surface and bacterial interactions, cells are initially guided to the surface by nonspecific long range, >50 nm, forces (e.g., gravitational, van der Waals, and electrostatic and hydrophobic forces) after which specific short-range, <5 nm, interactions (chemical, ionic and dipole, and hydrophobic interactions) weakly bind them to the substrate.^23−25^ The reversible binding is then strengthened by adhesion proteins and other surface polymeric structures resulting in permanent adhesion.^21,23^ On the other hand, mammalian cell adhesion relies on the adsorbed layer of protein formed during the initial contact between the bodily fluids and substrate.^26^ Eukaryotic cells will be attracted and bonded by physicochemical interactions; however, focal adhesion points will be formed through the adsorbed protein layer. Cell spreading will then be achieved through cytoskeleton filament contraction and secured by an equilibrium between tension of microfilaments and compression of microtubules,^26,27^ with subsequent migration, proliferation, and differentiation. Thus, it is clear that both prokaryotic and eukaryotic adhesion processes are heavily dependent on the physicochemical properties of both the cells and surface, pushing forward the biomaterial community to unearth correlations that can then be controlled to elucidate desirable device/tissue interactions.

Available literature can be found linking surface topology, wettability, or surface free energy of titanium surfaces to cell attachment with idiosyncrasies between bacterial and mammalian cell behaviors reported. Bacterial attachment is generally increased as the average surface roughness (*R*_a_) increases, although limited colonization can be achieved in the 0.5–1.5 μm range.^28−30^ On the other hand, previous studies have shown that the optimal *R*_a_ for mammalian and cell attachment may be around 1–1.5 μm;^31,32^ however, limited adhesion may be observed for higher *R*_a._(33) In contrast, other analyses seem to indicate that both organisms have enhanced proliferation in hydrophilic/high surface free energy^34,35^ or positively charged^27,36^ substrates. Nevertheless, the linkage between some of these fundamental properties, such as topology and contact angle (CA),^37^ and cellular responses has resulted in contradictory reports.^38−40^ For example, as the surface roughness reaches the nanoscale, bacterial adhesion would depend on both the cell membrane characteristics and variations in topological features (i.e., height, spacing or diameter of the main peaks).^41^ To partly overcome these difficulties, mathematical models have arisen, with particular attention paid to bacterial-solid surface interfaces such as the Derjaguin–Landau–Verwey–Overbeek (DLVO) and extended-DLVO (XDLVO) theories; however, the complexity and specificity of such interactions still affect their applicability.^21,36^

To support AM adoption, it is critical to understand the effect of base materials and postprocessing methods on cell interactions to encourage device longevity. The aim of this study was to establish relationships between the physicochemical properties of selective laser-melted Ti-6Al-4V samples on both bacterial and eukaryotic cell attachment. For this purpose, coupons were manufactured and postprocessed with high-degree polishing, passivation, and vibratory finish. Topology, chemistry, wettability, and surface ζ-potential were correlated with attachment and biofilm formation of *Staphylococcus epidermidis* and adhesion, proliferation, and mineralization of SAOS-2 osteosarcoma cells. These provide a new insight into the physicochemical interactions between AM materials and biological matter to drive the development of new custom devices.

## 2 Experimental Section

### 2.1 Sample Preparation

Circular coupons 10 mm in diameter and 3 mm in thickness were fabricated perpendicular to the building plane using a laser powder bed fusion additive manufacture system (RenAM 500 M, Renishaw PLC, United Kingdom). Ti-6Al-4V Grade 23 feedstock was supplied by Carpenter Additive (Carpenter Technology Corporation, US) with the powder in the size range of 15–53 μm. The RenAM 500 M operates in a modulated system which substitutes the scanning speed commonly defined in other systems through the exposure time and point distance. Thus, a layer thickness of 30 μm, a laser power of 200 W, a point distance of 55 μm, an exposure time of 50 μs, a hatch distance of 0.105 mm, a spot size of 70–75 μm, and four contours were selected to control the AM process. Samples were manually removed from the printing base, and all supporting structures were detached manually avoiding damage to the surface of study.

As-built samples were sequentially polished through a series of abrasive disks, MD-piano 120, 1200, 2000, and 4000 (Struers, UK) with water cooling, followed by mirror polishing with a hydrogen peroxide activated colloidal silica solution and an MD-chem pad (Struers, UK). A set of polished samples was passivated by immersion in a 10% HCl solution for 1 h. A second set was abraded with a vibratory finish machine containing an abrasive bonded inorganic ceramic media for the vibrofinishing process mixed with water and SX-1L following the manufacturer’s guidelines (Sharmic Engineering Ltd., UK) for 24 h. A rolled 316L stainless-steel sheet was used as a control sample throughout the study. After each treatment, all samples were cleaned in an ultrasonic bath with deionized water, acetone and, finally, ethanol for 10 min each, followed by air drying.

### 2.2 Physicochemical Analysis

#### 2.2.1 Surface Roughness and Chemical Evaluation

Surface finish was analyzed on the unsupported sides of the as-built samples using a noncontact profilometer (Infinite Focus G5 Optimax, Bruker Alicona, Austria) from an average of 10 measurements taken perpendicularly to the build direction using a ×20 lens. During profile acquisition, a Gaussian filter with cut off frequencies selected with compliance to ISO 4288-1996^42^ was selected. Metrology was complemented through surface imaging and chemical evaluation through a scanning electron microscope (JSM-6060, JEOL Ltd., UK) equipped with an energy-dispersive X-ray spectroscopy system, EDS (Inca 300, Oxford instruments, UK) unit.

#### 2.2.2 Wettability and Surface Free Energy

The ability of a liquid to wet the selected surfaces was evaluated through CA measurements using the sessile drop technique (OCA 25 Optical CA system, DataPhysics, Germany). Two microliters of four different liquids, namely, deionized water, ethylene glycol, glycerol, and dimethyl sulfoxide (DMSO), were deposited onto the surfaces and, after 5 s of contact, images were recorded. The resulting CA was calculated as the average of three independent measurements for each sample. Surface free energy was calculated using the Lifshitz–van der Waals/Acid–Base (LW/AB) theory and surface tension of the specified liquids obtained from the literature (Table 1).^43^

**Table 1:** Components of the Surface Tension Used in the Present Study (mJ/m^2^)^a^

liquid	γ_t_	γ^LW^	γ^AB^	γ^+^	γ^–^	γ^AB^/γ^LW^
deionized water	72.8	21.8	51	25.5	25.5	2.3
ethylene glycol	48	29	19	1.92	47	0.7
DMSO	44	36	8	0.5	32	0.2
glycerol	64	34	30	3.92	57.4	0.9

a Columns represent the total, γ_t_, nonpolar or Lifshitz–van der Waals, γ^LW^, polar or acid–base, γ^AB^, electron acceptors, γ^+^, donors, γ^-^, components, and polarity γ^AB^/γ^LW^.

#### 2.2.3 ζ-Potential

To evaluate the differences in SZP, suspensions of aliphatic amine latex beads (Life Technologies) were prepared in phosphate buffer saline. This solution contained 1.37 mM NaCl, 27 μM KCl, and a total phosphate concentration of 100 μM at pH = 7.4. SZP determinations were carried out using the tracer particle method,^44,45^ through a SZP cell (Malvern Instruments, UK). Briefly, the tracer particle mobility in an alternate current field is probed at varying displacements from the surface under study (250, 375, 500, and 1000 μm), yielding determinations of the apparent SZP value of tracer particles at each displacement. The greater the distance from the surface, the smaller the effect of electro-osmotic flow, so that at a sufficiently large distance the mobility is determined only by electrophoretic migration, yielding the intrinsic SZP of the tracer particles. From the obtained apparent SZP values, a linear extrapolation to the intercept at zero displacement can be used to estimate the SZP of the surface using the equation ζ_Surface_ = ζ_Tracer_ – intercept. These measurements were obtained through three independent measurements.

### 2.3 Bacterial Proliferation and Imaging

Colonization of AM surfaces with a clinically relevant implant-infection bacteria was studied by culturing *S. epidermidis*, ATCC 12228, biofilms following the protocol of Christensen et al.^46^ Briefly, surfaces were degreased with acetone, sterilized by autoclaving, then immersed in pure ethanol for 5 min, dried under UV light for another 5 min, and kept sealed for more than 24 h before testing. The lamp emits predominantly 240 nm UV light, and all samples were situated 100 mm from the source. An overnight culture of *S. epidermidis*(47) in sterile Mueller Hinton broth was diluted to ∼10^3^ CFU/mL, and 1 mL was inoculated onto samples placed in a 24 well plate. Plates were kept in an orbital incubator for 24 h at 37 °C, washed gently three times with Dulbecco’s phosphate buffered saline (DPBS), and fixed in a 2.5% glutaraldehyde solution for 1 h. Crystal violet was used to estimate the bacterial biomass formed on the surfaces similarly to the method used by O’Toole.^48^ Briefly, each surface was covered with 200 μL of a 0.5% crystal violet solution for 5 min. Excess staining was removed by washing each sample in DPBS (Sigma-Aldrich, UK) and fully dried in an incubator at 37 °C. Then, each sample was immersed in 1 mL of methylated spirit for 2 h, and absorbance was quantified at 590 nm wavelength using a TECAN Spark plate reader (Tecan Trading AG, Switzerland). All experiments were repeated, and results are the average of three independent measurements.

To complement the biomass analysis, one sample per condition was visualized using microscopy. Each sample was washed gently three times with DPBS, fixed with 2.5% glutaraldehyde in DPBS for 1 h, dehydrated with a series of ethanol and deionized water dilutions (10 min sequentially each in 20, 30, 40, 50, 60, 70, 90, 95, and 100%), treated with hexamethyldisilazane, and dried overnight. Before imaging, each sample was mounted on an aluminum stub, gold-sputtered, and imaged using an acceleration voltage of 15 kV in a Zeiss EVO M10 microscope (Carl Zeiss GmbH, Germany). For confocal fluorescence imaging, one sample per condition was washed gently three times with DPBS and stained with 200 μL of a DAPI and fluorescein isothiocyanate-conjugated wheat germ agglutinin, WGA (Vector laboratories, UK) solution and incubated for 30 min. Imaging was carried out using a ZEISS LSM 710 confocal microscope (Carl Zeiss GmbH, Germany) at ×10 magnification, and coverage was calculated using ImageJ (National Institute of Health, version 1.53a).

### 2.4 DLVO and XDLVO Models

Physical interactions caused by electrostatic, van der Waals, and Lewis acid–base forces were estimated through the DLVO and XDLVO models, following the method proposed by Wu et al.^49^ All calculations were performed in MATLAB R2021a version 9.10.0.1669831 (MathWorks Inc., USA).

### 2.5 Cell Culture

Cellular interactions with the postprocessed samples were analyzed using a bone-forming human osteosarcoma cell line (SAOS-2, P12). Before seeding, all samples were degreased and disinfected following the protocol previously described in Section 2.3. Samples were inoculated with 2 × 10^4^ osteoblastic cells and incubated for 40 min (37 °C and 5% CO_2_) to allow initial cell adhesion. Subsequently, 1 mL of Dulbecco’s modified Eagle’s medium (10% fetal bovine serum, 12% l-glutamine, and 1% penicillin/streptomycin) was added, and cells were cultured at 37 °C and 5% CO_2_. After 3 days of growth, media were exchanged with osteogenic media, and basal media were modified with 50 μg/mL ascorbic acid, 10 mM β-glycerophosphate, and 100 nM dexamethasone (Sigma-Aldrich), with further changes in media every two to three days for a total of 28 days.

Prior to confocal imaging, three samples per condition and contact timeframe (2.5 and 24 h) were washed gently with DPBS, fixed in a 4% solution of paraformaldehyde for 10 min, and incubated at 37 °C and 5% CO_2_ for 10 min in 0.1% Triton (AnaSpec Inc., US). Samples were then treated with 200 μL of a mixture of Phalloidin 488 and DAPI in DPBS and further incubated for 20 min. These were then moved into plastic confocal dishes and submerged in DPBS, and images were taken using a Leica TSC SP8 (Leica Microsystems Ltd., UK) with excitation and emission lines selected following the manufacturer recommendations. Three large and three high-magnification images per sample, condition, and timepoint were randomly taken at the center of the sample where the initial cell seeding was performed.

Coverage was calculated using ImageJ (National Institute of Health, version 1.53a) by splitting the channel corresponding to the actin dye and thresholding with Huang’s filter for the low-magnification images. Illuminated and total pixels were counted to estimate cell coverage with results being the average of nine images with standard deviation reported. To analyze the morphology of high-magnification images, a custom script was prepared in MATLAB R2021a. The green channel was first split, and an approximate region containing the cell to be analyzed was manually selected. The background was masked, brightness maximized, and filled using the inbuilt Image Processing Toolbox, and results were obtained from at least 100 cells. Circularity, aspect ratio, and roundness were defined by eqs 1–3
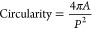
1
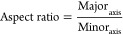
2
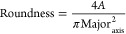
3where *A* is the area of the cell, *P* is the perimeter or the cell, Major_axis_ is the major axis of the best fit ellipse for the measured cell, and Minor_axis_ is the minor axis of the best fit ellipse for the measured cell.

Metabolic activity was assessed after 1, 3, 7, and 14 days of culture by transferring the samples to a new 24-well plate, pouring 1 mL of a 10% mixture of fresh media and Alamar Blue (Thermofisher scientific), following incubation at 37 °C and 5% CO_2_ for 4 h. One hundred microliters of the media and Alamar Blue mixture per sample were recovered, and fluorescence was measured with 560 and 590 nm excitation and emission wavelengths, respectively. Samples were washed with DPBS, and fresh media were added.

To estimate the initial osteogenic potential of the treated surfaces, alkaline phosphatase (ALP) content was measured using a SensoLyte pNPP Alkaline Phosphatase Assay Kit (AnaSpec Inc., US) after 1, 3, 7, 14, 21, and 28 days of culture. Shortly, samples were washed in 1 mL of 1X buffer, treated with 1 mL of Triton-X solution, and incubated for 1 h (37 °C, 5% CO_2_, 95% air). Lysation was carried out by freezing and thawing all testing coupons from −80 to 37 °C three times and, after each thawing, samples were set in an ultrasound bath for 20 min, and a pipette tip was used to scratch each surface. Finally, 25 μL of the recovered lysate was diluted with 25 μL of 1X buffer, treated with 50 μL of pNPP solution, and incubated for 15 min (37 °C, 5% CO_2_, 95% air), and absorbance was measured at 405 nm using a TECAN Spark plate reader (Tecan Trading AG, Switzerland). ALP standards were prepared using the ALP solution provided in the kit. Results were the average of three independent measurements for each sample.

To normalize ALP content, DNA quantification was performed by taking 10 μL of the ALP lysate in a 96-well plate and adding 90 μL of 1X TE buffer (Thermo Fisher scientific, USA). Then, 100 μL of PicoGreen solution (Thermo Fisher scientific, USA) were added, and plates were incubated for 5 min at room temperature protected from light. Fluorescence was measured at 480 nm excitation and 520 nm emission wavelength, respectively, using a SPARK plate reader (Tecan Trading AG, Switzerland). DNA standards were prepared with a preprepared DNA standard solution (Thermo Fisher scientific, USA), and all results were the average of three independent measurements for each sample.

Calcium deposits were assessed after 7, 14, 21, and 28 days of culture by fixing the surfaces in 4% paraformaldehyde for 30 min, rinsing in 10 mM DPBS, and staining with a 2% alizarin red (AR) solution for 20 min. Excess staining was removed by washing three times in deionized water, and samples were moved to a new plate and left to dry overnight. AR stain was recovered by immersion in a 10% cetylpyridinium chloride (CPC) solution for 1 h at room temperature with samples protected from light and set in a plate shaker at 40 rpm. Absorbance was measured with a TECAN Spark plate reader (Tecan Trading AG, Switzerland) using 570 nm. Scanning electron microscopy (SEM) imaging was performed through the same method previously discussed in Section 2.2. All results were calculated as the average of three independent samples with standard deviation reported.

Qualitative assessment of calcium and phosphate deposition was performed using X-ray fluorescence (XRF). Briefly, samples treated following SEM fixation and dehydration were carbon-coated and analyzed in a M4 Tornado micro-XRF at 50 kV, 400 μA, with a spot distance of 30 μm and 1 ms dwell time, in serpentine stage mode for five cycles.

To analyze the ability of the selected surfaces to adsorb proteins, sterilized samples were cultivated in Dulbecco’s modified Eagle’s medium (10% fetal bovine serum, 12% l-glutamine, and 1% penicillin/streptomycin) for 24 h. Samples were recovered, washed twice in DPBS, and further analyzed using a Pierce BCA protein assay kit (Thermofisher scientific) following the manufacturer’s guidelines. Shortly, each coupon was placed in a 24-well plate and 1 mL of working solution (50:1 Reagent A:B) and incubated at 37 °C for 30 min. After cooling to RT, 100 μL per sample were collected, and absorbance was measured at 562 nm wavelength using a TECAN Spark plate reader (Tecan Trading AG, Switzerland).

Variations in extracellular matrix production were assessed through collagen staining with picrosirius red after 7, 14, and 21 days of culture. Briefly, cultured samples were washed twice in PBS and fixed in 4% paraformaldehyde for 30 min and stained with a Picro-Sirius Red Solution (ScyTek Laboratories, Inc., USA) for 1 h at room temperature and protected from light. Excess dye was removed by washing in 0.5 M acetic acid followed by distilled water and air-drying. Then, samples were treated with 0.5 M sodium hydroxide to elute the bound dye, and absorbance was read at 590 nm using a SPARK plate reader (Tecan Trading AG, Switzerland).

### 2.6 Statistical Analysis

All statistical analyses were performed with SPSS (IBM Corp. Released 2015. IBM SPSS Statistics for Windows, Version 23.0). Prior to any test, the similarity of variances between groups was studied through Levene’s test. If similarity between variances could not be rejected, an ANOVA-I test followed by Tukey’s post hoc with an alpha level of 0.05 was used. In contrast, a rejection of similarity between variances resulted in a Welch’s test and Games-Howell’s post hoc as suggested by Fiend and Miles.^50^

## 3 Results and Discussion

### 3.1 Physicochemical Analysis

**Figure 1:**
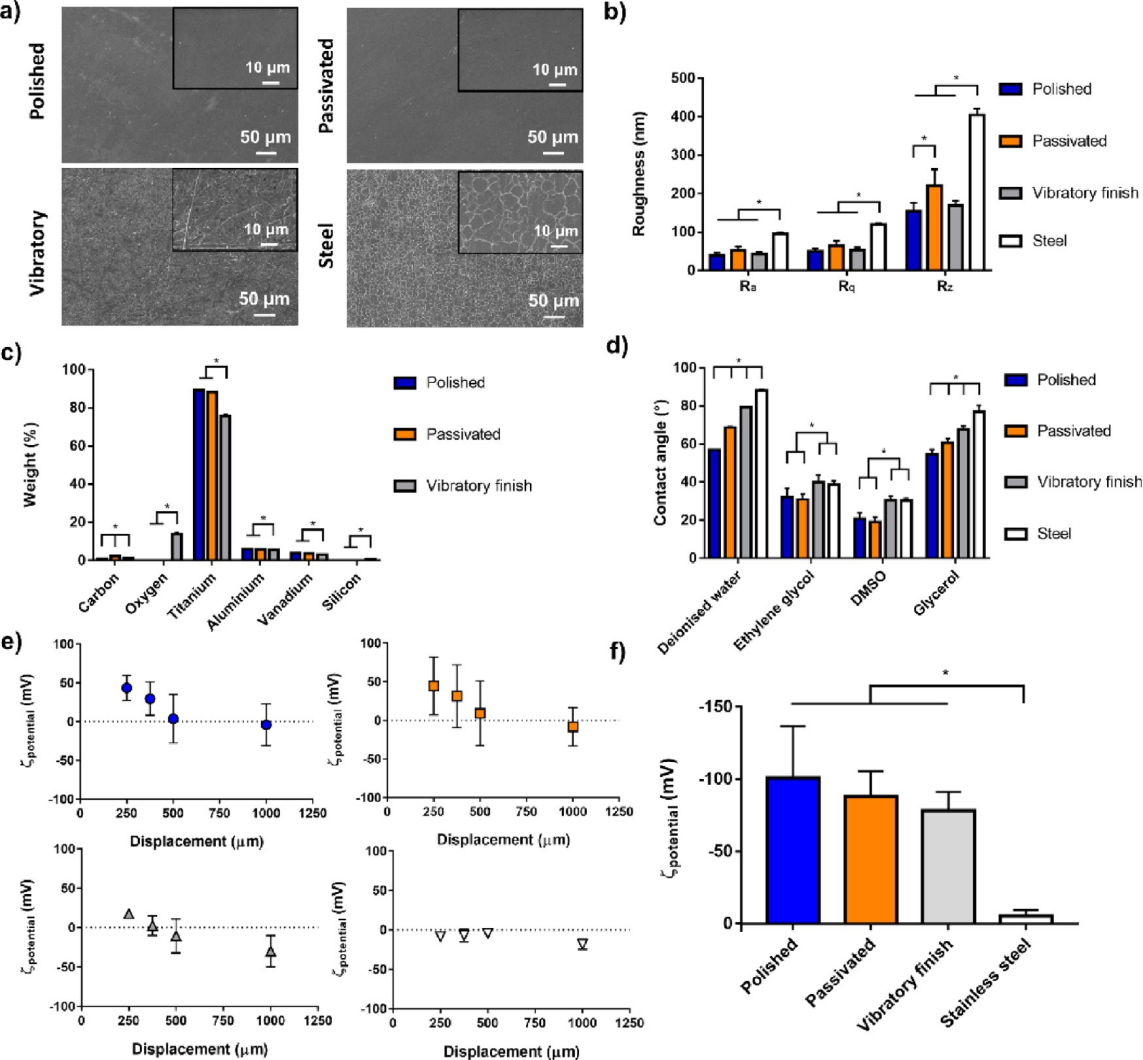
Physicochemical analysis of metallic surfaces including (a) surface SEM images, (b) measured arithmetic mean height (*R*_a_), root mean squared height (*R*_q_) and maximum height of profile (*R*_z_), (c) chemical analysis through energy-dispersive X-ray spectroscopy, (d) CA for four liquids with different polar and dispersive free energy components, (e) apparent ζ-potential of aliphatic amine latex tracer particles versus surface displacement, and (f) surface ζ-potential for all the processed surfaces, where * indicates *p* < 0.05 between groups.

Postprocessing of the AM titanium samples led to smooth surfaces (Figure 1a,b), with a mean surface roughness (*R*_a_) ranging between ∼40 and ∼50 nm. Both polished and passivated samples were characterized by the limited presence of imperfections, suggesting a highly dense base material. In contrast to the unblemished substrates, vibratory finishing led to a visibly scratched surface as a consequence of the interaction between the hard-ceramic media and AM surface. Despite these apparent dissimilarities, topographical measurements indicate that no statistical difference (*p* > 0.05) can be observed for the average and square root roughness (*R*_q_) of the titanium samples. More substantial variations in composition could be observed through EDS analysis (Figure 1c). Passivation of the AM samples may have resulted in a slight increase, ∼1.5%, in carbon content from that displayed in the polished surface, but no substantial variation in oxygen could be noticed. This was not the case for the vibratory finished samples which exhibited 13.8 ± 0.9% of O alongside small traces, 0.5%, of silicon possibly resulting from abrasive media cross contamination.^51^ Compared to these alloys, the 316L stainless-steel is dominated by large grains protruding from the surface resulting from the rolling process.^36^ These structures led to a relatively coarse surface with an average *R*_a_ of 95.5 ± 3.8 nm and a maximum peak to valley height of the profile of 403.9 ± 17.4 nm.

CA measurements indicated that all surfaces were hydrophilic (CA < 90°), although the titanium alloys revealed a steep increase in wettability between the polished, 56.42 ± 0.74°, passivated, 69.06 ± 1.04° and vibratory finished, 79.85 ± 1.14° surfaces when compared to the stainless-steel, 89.9 ± 1.4° control. A similar trend and range of CA measurements are followed through the sessile assay conducted with glycerol although, more interestingly, the two less polar liquids, namely, ethylene glycol and DMSO subdivide the tested samples into two clearly differentiated groups (polished/passivated and vibratory finished/stainless-steel). These trends result in a generalized decrease in total and polar surface free energy (Table 2) as the CA increases, with similar nonpolar components in all samples analyzed. In contrast, further subdivision of the polar component into electron donor and acceptor constituents resulted in the former being almost unchanged, with the latter highly decreasing for the vibratory finish and stainless-steel control.

**Table 2:** Components of the Surface Free Energy of the Selected Materials Calculated Following the LW/AB Theory (mJ/m^2^)^a^

	γ_t_	γ^LW^	γ^AB^	γ^+^	γ^–^	γ^AB^/γ^LW^
polished	40.9	27.8	13.1	1.7	24.8	0.5
passivated	39.3	30.7	8.7	1.7	11.2	0.3
vibratory finish	34.3	28.6	5.7	1.8	4.5	0.2
stainless-steel	32.1	30.1	2.0	1.9	0.5	0.1

a Columns represent the total, γ_t_, nonpolar or Lifshitz–van der Waals, γ^LW^, polar or acid–base, γ^AB^, electron acceptors, γ^+^, donors, γ^-^, components, and polarity γ^AB^/γ^LW^.

To fully understand the role of nonspecific forces on surface–cell interactions, all previous measurements were complemented by the evaluation of surface ζ-potential, SZP. All metallic surfaces presented negative SZP values (Figure 1e,f), with a slight decrease between polished, passivated, and vibratory finished Ti-6Al-4V, −100.7 ± 35.8 mV, −88.0 ± 17.5 mV, and −78.3 ± 12.9 mV, respectively. Nevertheless, no statistical difference was observed between these groups (*p* > 0.05), with only material suggested to be the main driver behind variations in SZP. Previous reports of Ti surface potential are scarce, although SZP values between −60 and −80 mV for Ti-6Al-4V can be found in the literature,^52^ suggesting that processing via AM results in a similar surface potential than conventional techniques. In the case of stainless steel, the range of SZP values reported is broad (+30 to −100 mV) and dependent on finish, roughness, and cleaning treatments as well as on ionic strength and pH of the solution used during measurements.^53,54^ SZP values for bare stainless steel in Figure 1f are negative, in agreement with most of the reported SZP values at pH 7 in the literature.^49,52,55^

Figure 1 indicates that all postprocessing methods applied affected the topology and chemistry of the samples, resulting in an increase in hydrophobicity and reduced surface free energy. Available literature in postprocessing of additive manufactured parts is focused on the physicochemical analysis of microrough samples^16,17^ with limited manuscripts understanding their effect in the sub-micro- and nano-scales. Nevertheless, reports on the physicochemical properties of mechanically or electropolished titanium alloys indicate that the CA, 60–70°, and surface free energies, ∼40 mJ/m^2^, are in agreement with those presented in this study.^56,57^ The similarities in topology between the passivated and polished samples suggest that variations in wettability were driven by chemical modification. In this regard, it is recognized that the hydrophilic nature of titanium-based surfaces is caused by its protective oxide layer.^35,58^ Kubies et al.^57^ showed a reduction in the CA on titanium alloys etched with hydrochloric acid as a result of a hydrated TiO_2_ layer, contrasting with the increase noticeable in Figure 1d. EDS measurements indicated that this postprocessing did not increase the presence of oxygen-rich groups but resulted in more adhered carbon-rich species. Thus, it seems likely that the reduction in hydrophilicity is a consequence of inorganic anions or organic hydrocarbon groups, which rapidly bind to high-energy titanium surfaces.^35^ A similar chemically driven modification may explain the further increase in the CA experienced by the vibratory finished samples; however, it is necessary to mention that the valleys formed on the surface could have enhanced this effect. The valleys formed by the ceramic abrasive may have entrapped air between the solid liquid interface following the Cassie–Baxter theory, instead of a complete wetting mode as proposed by Wenzel’s model.^59,60^ Nevertheless, the contribution of this effect may be limited, as suggested by the similarities in surface roughness revealed in Figure 1b. In contrast, both chemical and topological effects should impact the control stainless-steel, in line with the analysis of Bakterij et al.^36^ and Estrada-Martínez et al.^61^

### 3.2 Bacterial Response to Surface Properties

**Figure 2:**
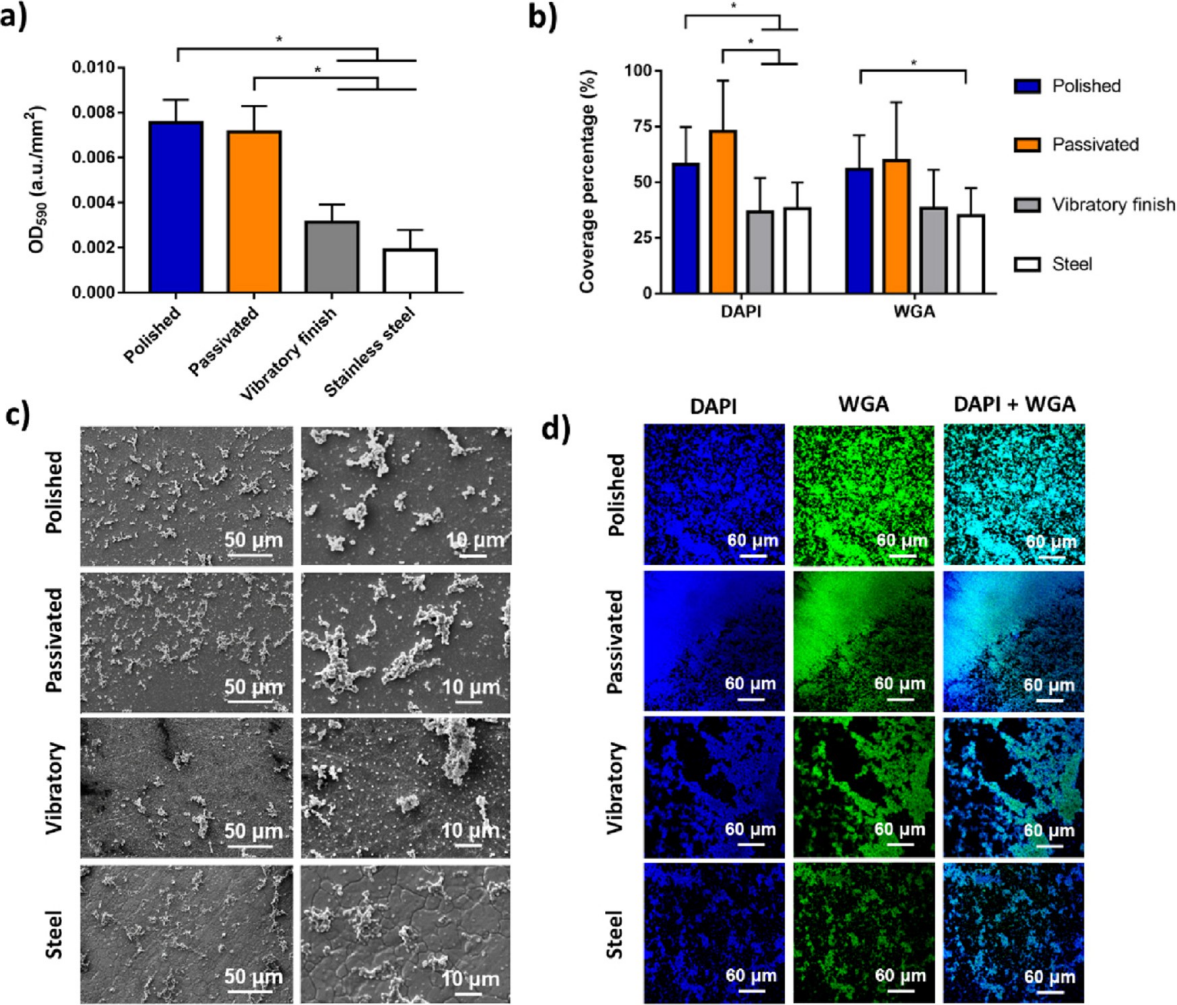
*S. epidermidis* response to postprocessed surfaces after 24 h of inoculation including (a) biomass quantification through crystal violet staining and (b) DAPI and fluorescein conjugated wheat germ agglutinin coverage analysis alongside (c) SEM micrographs and (d) confocal images, where * indicates *p* < 0.05 between groups.

The ability of *S. epidermidis* to colonize and produce a biofilm was highly dependent on the selected postprocess and material (Figure 2a,b). Total biomass attached to the engineering surfaces was subdivided in two clear groups with both polished and passivated samples supporting greater biomass production (absorbances up to 0.008 a.u./mm^2^), contrasting with lower values obtained for the vibratory finished and stainless-steel samples (absorbance of 0.003–0.002 a.u./mm^2^). A similar trend was confirmed by confocal staining, although statistical differences were dependent on the stain used. Coverage calculated through nucleic acid staining (directly reflecting cell numbers) followed similar trends to those obtained through crystal violet staining. In contrast, glycoprotein staining through WGA (reflecting matrix production) showcased more subtle differences, with only the polished sample statistically, *p* < 0.05, different from stainless-steel. The data suggest that the main effect of the postprocessing and material selection has been the reduction or enhancement of bacterial attachment and growth with minimal to no influence in early biofilm formation. These differences can be clearly appreciated in both SEM and confocal images (Figure 2c,d). For both polished and passivated samples, numerous small aggregations of bacterial cells can be seen in the confocal images. A lower density of larger aggregates is noticeable for both vibratory finished and stainless-steel coupons with large voids between these structures. SEM micrographs are prepared through a more complex and destructive process which may explain the differences in coverage between techniques; nevertheless, the variations in number of cells seen is consistent with biomass measurements.

The ability of bacteria to colonize and proliferate on surfaces is highly dependent on the physicochemical properties of the tested material and the nature of the microorganism tested. In the case of surface roughness, topology and dimensions of the surface features are of special relevance in the micro and nano scale where the bacterial size is of similar magnitude to the aforementioned parameters.^36,39^ Research on the relationship between surface features and bacterial size has generally shown that ultrasmooth and submicron finishes tend to reduce bacterial attachment, while microtopologies tend to promote it.^3,22,23^ Two examples emphasizing the importance of the surface roughness scale can be found in the work of Taylor et al.^62^ and Jhong et al.^28^ who have showcased the higher affinity for bacterial attachment when an initial ultrasmooth surface, 10 nm, becomes coarser, 1240 nm, and similar in size to that of the bacteria analyzed. Nevertheless, controversy still exists with reviews of the available literature reporting conflicting results.^3^ In the present study, both polished and passivated samples showcase an ultrasmooth surface. These display an *R*_a_ one scale of magnitude smaller than the average diameter of *S. epidermidis* cells (0.5–1 μm) with no visible imperfections that could act as reservoirs for bacterial attachment. On the other hand, the vibratory finish and stainless-steel samples showcase imperfections in the form of peaks and valleys that can act as promoters for bacterial attachment. However, the present study indicates the opposite which, coupled with the similarities in surface finish between samples, suggest a major role of other physicochemical properties on bacterial proliferation. It must be mentioned that numerous mechanisms influence bacterial attachment in the nanoscale, namely, chemical gradients, physicochemical forces and cell membrane deformation, and configuration.^41^*S. epidermidis* is a Gram-positive bacteria surrounded by a protective peptidoglycan layer,^63^ meaning that it has a limited ability to deform to comply with rough surfaces. In the case of both vibratory finished and stainless-steel surfaces, the depth and spatial configuration of the valleys limit the available contact area which, coupled with the stiff outer membrane, may have influenced the attachment of *S. epidermidis*.^39,64^

Alongside surface topography, the roles of wettability and surface free energy in biomaterial and cell interactions have to be recognized. Great effort has been made by the biomaterials community to understand the role of hydrophilic (CA < 90°), hydrophobic (CA > 90°) and superhydrophobic (CA > 150°) materials in cell attachment.^65^ It has been shown that, generally, increasing CAs tend to reduce bacterial adhesion.^28,66,67^ Thus, it seems reasonable to infer that increased hydrophobicity of the vibratory finished and stainless-steel samples may explain the reduced bacterial biomass observed in Figure 2. Nevertheless, the main mechanism explaining hydrophobicity in the Cassie–Baxter theory relies on air entrapment in topological features.^68^ The stability of air inside valleys is not completely understood which, coupled with contradictory reports^69^ highlight that wettability may not be a clear indicator of bacterial attachment. In fact, other researchers have shown that its influence may be dependent on the wettability of both surface and bacterial strain, leading to a rule of thumb for which hydrophobic bacteria adhere predominantly to hydrophobic surfaces and vice-versa.^21,70,71^ Available literature regards *S. epidermidis* as a hydrophilic species;^72^ thus, its affinity with water would result in a surrounding layer of adsorbed water, limiting further contact and adhesion to hydrophobic materials, as proposed by van Loosdrecht et al.^25^ At the same time, there appears to be a remarkable correlation between bacterial proliferation (Figure 2) and acid–base, γ^AB^, and electron donor, γ^–^, components of the surface free energy (Table 2). While it has been hypothesized that higher values of total surface free energy could led to higher probabilities of attachment,^73^ the studies of Boulange-Petermann et al.^74^ and Sardin et al.^75^ suggest that this correlation is mostly dependent on the balance between polar and nonpolar components. Based on the results obtained in the present study, it seems probable that *S. epidermidis* adhesion and proliferation are enhanced on polar and less hydrophobic materials, which has also previously been suggested by Renner et al.^76^ At the same time, it must be mentioned that specific materials may need further consideration. UV-irradiated titanium alloys have recently shown their ability to retain their antimicrobial properties after exposure.^77,78^ While this may have influenced the current study, it should be recognized that irradiated stainless steel has revealed a reduced effect on other Gram-positive bacteria with low exposure times.^79^ In this case, the bacterial growth observed in Figure 2 and its comparison with the titanium substrates suggest a limited effect of UV irradiation.

**Figure 3:**
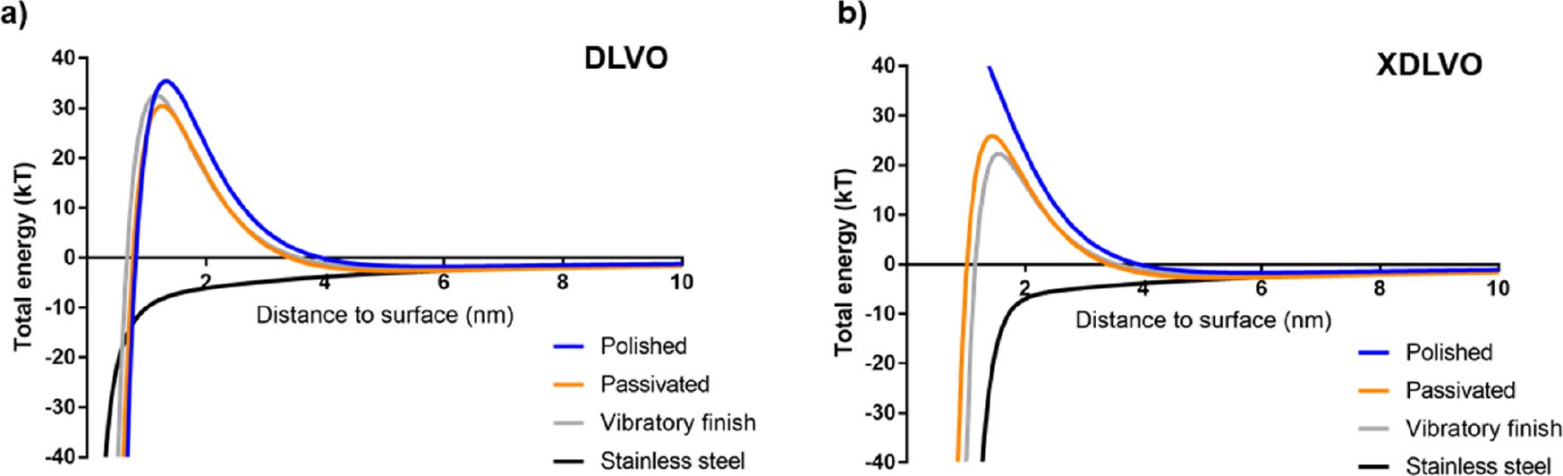
Total interaction free energy between a *S. epidermidis* cell and the analyzed surfaces using the (a) Derjaguin–Landau–Verwey–Overbeek, DLVO, and (b) extended-DLVO, XDLVO, colloidal theories.

*S. epidermidis* cells are normally considered to have a net negative surface charge (i.e., -10 mV),^49^ which coupled with the negative SZP (Figure 1f) indicate that a net repulsive force would prevent bacterial adhesion more effectively on titanium surfaces than stainless steel. However, bacterial colonization was more substantial on polished and passivated titanium substrates, which could indicate that attractive van der Waals or acid–base forces may have been predominant on these surfaces. When the attractive energy arising from Lifshitz–van der Waals forces and the repulsive electrostatic interactions of a bacterial cell approaching a surface are considered through the classical Derjaguin–Landau–Verwey–Overbeek, DLVO, theory (Figure 3a),^80,81^ it can be seen that only stainless steel presents an attractive net negative energy. In contrast, the analyzed titanium surfaces would reveal an initial attraction followed by a repulsive maximum around 1 and 2 nm with an energy barrier ∼30kT, which should be overcome to enable adhesion. If the Lewis acid–base interaction is considered through the extended-DLVO theory (Figure 3b),^80,81^ there is a slight reduction in this energy barrier for the passivated and vibratory finish, indicating an attractive nature of this force. However, the high electron donor component of polished Ti-6Al-4V and *S. epidermidis* has resulted in a positive characteristic decay length, Δ*G*_ho_^AB^, and subsequent repulsive energy with a dramatic increase in the energy barrier, ∼1940 kT. Both models support the dominance of repulsive forces for the bacteria species considered, which contrast with the results obtained in this manuscript. Previous studies have tried to explain bacterial interactions through both mathematical models with various degrees of success;^82,83^ however, it is becoming clearer that these theories are limited. The cell wall is not a perfectly rigid body instead able to adapt to the substrate, while the surface is not a perfectly flat plane, both of which are essential assumptions in these theories.^49,84^ Similarly, most bacterial species possess fimbria and other appendages, which have been suggested to create adhesion points by overcoming the surface energy barrier,^49,85^ while only nonspecific forces are considered.^80^ Thus, it is clear that more complex theories are necessary to account for surface bacterial interactions.^82^ Similarly, the current manuscript focuses on early surface interactions of *S. epidermidis*, nevertheless, and given the importance of biofilm formation on medical devices, follow up studies centered on different bacterial strains with various biofilm forming abilities should be performed.

### 3.3 Biological Response of Eukaryotic Cells

**Figure 4:**
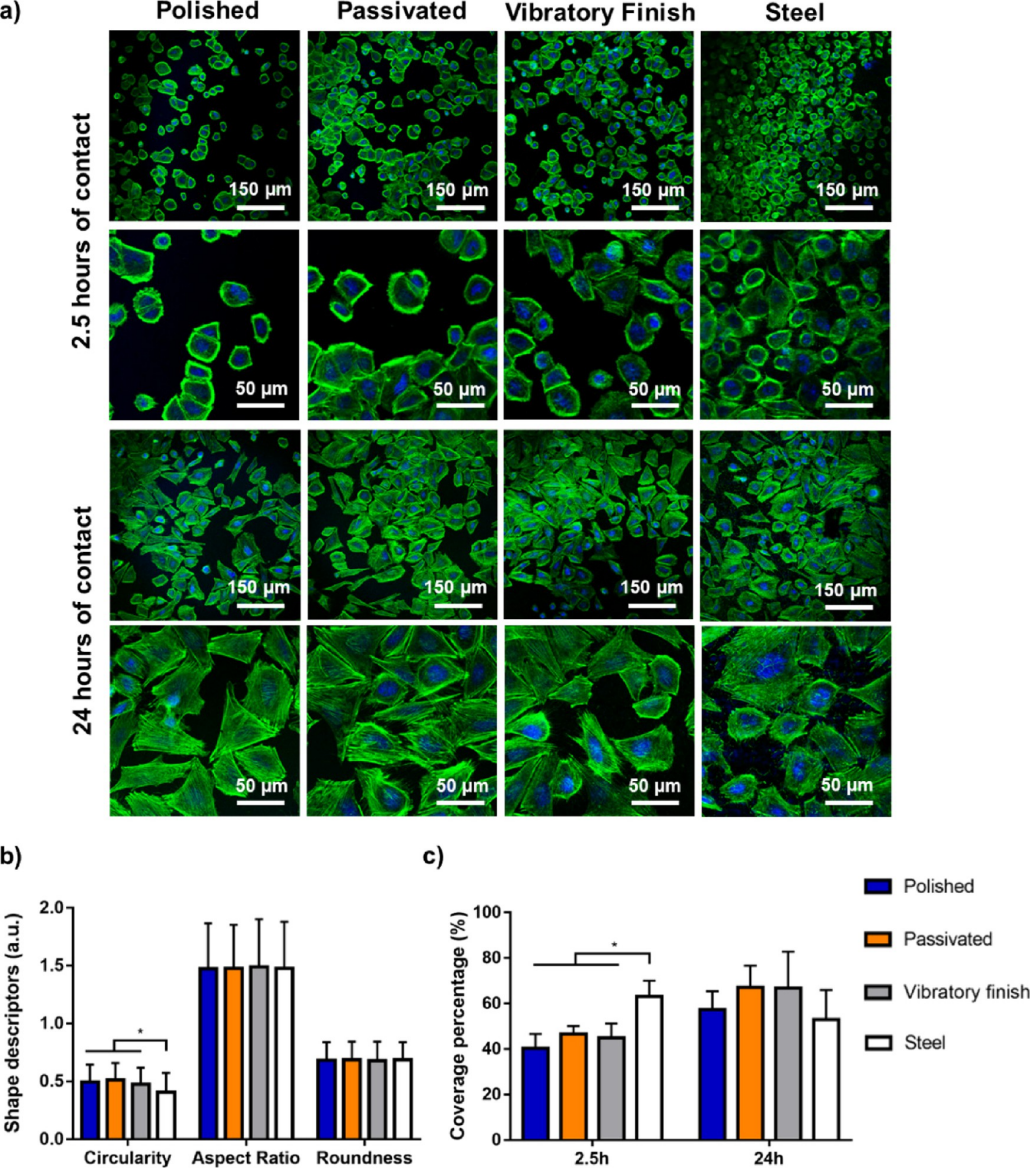
Early attachment of human osteosarcoma cell line SAOS-2 on postprocessed surfaces including (a) actin and DAPI confocal staining after 2.5 and 24 h of seeding, (b) shape analysis (circularity, aspect ratio, and roundness) after 2.5 h of contact, and (c) coverage percentage of actin for both timeframes considered, where * indicates *p* < 0.05 between groups.

The influence of surface treatment on early attachment to mammalian cells was initially analyzed through confocal staining of SAOS-2 human osteosarcoma cells (Figure 4a). After 2.5 h of contact, all Ti-based surfaces revealed large clusters of cells divided by areas with limited cell coverage while stainless steel showcased a homogeneous layering. At this stage, the actin cytoskeleton seems to be highly packed into a generally ovoidal or irregular contour, although slight differences could be observed through morphological analysis (Figure 4b). Average results indicate that cells deposited on stainless-steel tend to have a significantly (*p* < 0.05) lower circularity, 0.41 and 0.5, respectively, suggesting slightly superior spreading on this surface. Nevertheless, the similarities in both aspect ratio and roundness seem to indicate that these differences may be minimal with a general elongation in one axis of the cell. Further contact time led to an increase in surface coverage, reaching confluent levels after 24 h, similar to those displayed on stainless-steel (Figure 4c). In all cases, the previously bundled structures were more open with long actin filaments leading to mostly ovoid or triangular shapes. Thus, the limited differences in early attachment were mostly caused by material selection rather than postprocessing, which quickly became negligible after a single day of contact. The decrease in cell coverage for stainless steel between 2.5 h (63.16 ± 6.89) and 24 h (52.89 ± 13.06) is noted and may have resulted from cell migration during the early stages of surface interaction.^86^

**Figure 5:**
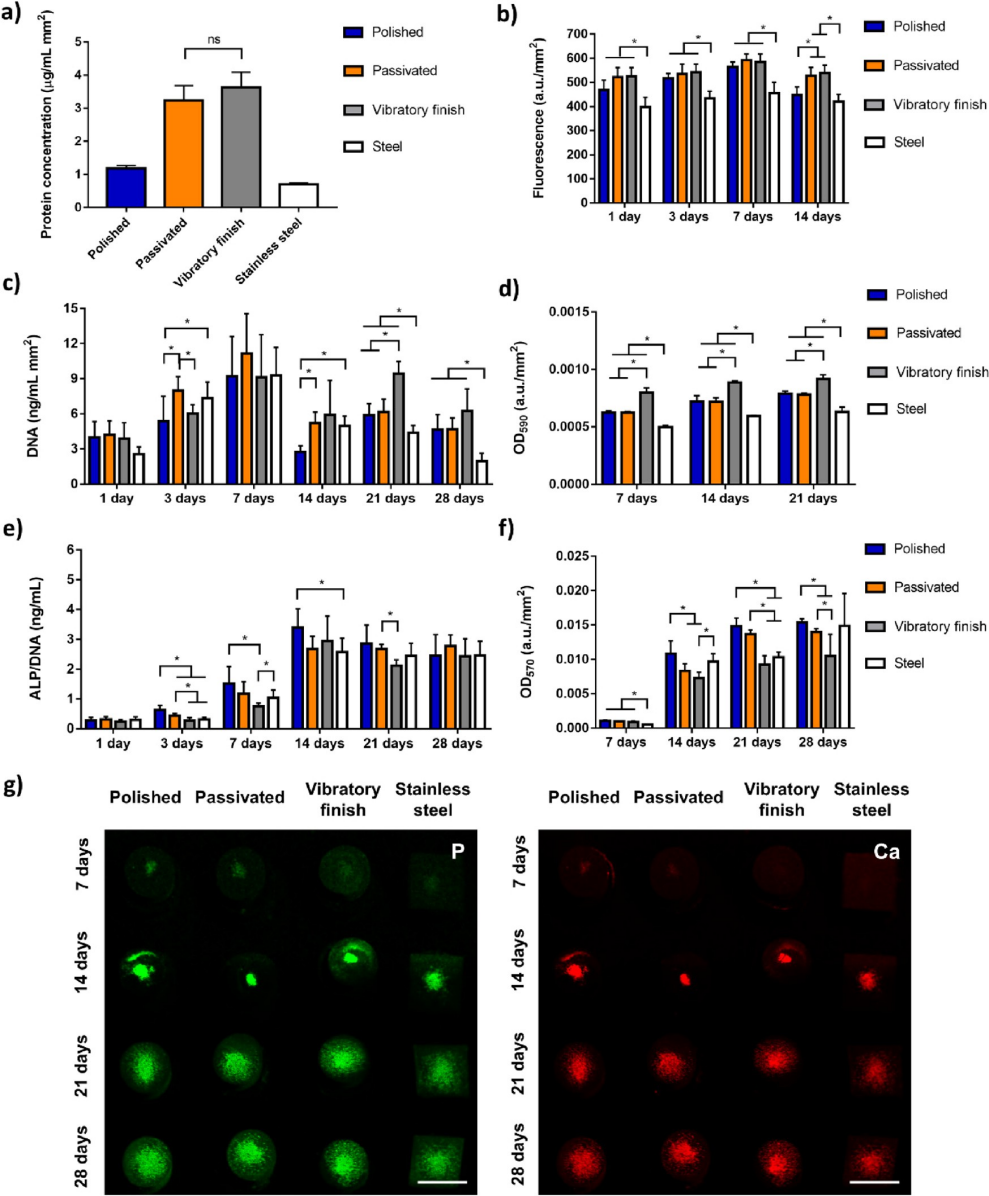
Biological evaluation of the selected surfaces analyzed through (a) total protein adsorption after 24 h of cultivation in 10% FBS DMEM media and viability and mineralization of human osteosarcoma cell line SAOS-2 including (b) metabolic activity assessed by Alamar Blue (c) DNA content, (d) collagen production, (e) internal ALP content normalized with DNA, (f) calcium deposits measured by Alizarin red staining, and (g) XRF elemental maps for calcium and phosphate (scale bar = 10 mm), where* indicates *p* < 0.05 between groups and ns *p* > 0.05.

To further analyze the biological response of SAOS-2 cells to postprocessing and substrate material, protein adhesion, metabolic activity, DNA content, collagen production, and mineralization were analyzed (Figure 5). An affinity for protein adsorption could be observed for the passivated, 3.24 ± 0.44 ng/mL mm^2^, and vibratory finished, 3.63 ± 0.46, samples (Figure 5a), contrasting with the similarities in metabolic activity of seeded osteosarcoma human cell line SAOS-2 (Figure 5b). All groups showed a general increase in activity after 3 and 7 days of cell seeding, followed by a decrease in metabolism after 14 days. Limited statistical differences could be observed, mostly focused on the lower normalized fluorescence of stainless steel, *p* < 0.05, which could indicate lower coverage of the sample. The amount of recovered DNA (Figure 5c) steadily increased in all groups over the first week of cultivation, suggesting that the cell number may have increased equally between groups in accordance with the metabolic activity and early cell seeding (Figure 4). In contrast, DNA content generally decreased to levels similar to those observed at day 3 after 2 weeks of seeding, which are maintained until the end of the study. Statistical analysis revealed that during the early stages of proliferation, day 3, both passivated and stainless steel displayed significantly higher DNA content, *p* < 0.05. However, further cultivation suggested that vibratory finishing encouraged cell proliferation, with stainless steel showing the lowest DNA content from all surfaces considered.

Collagen production steadily increased in all groups considered (Figure 5d), albeit vibratory finishing and stainless steel revealed significantly higher and lower, respectively, content throughout days 7, 14, and 21. Normalized ALP (Figure 5e) was minimal at day one, slightly raising during day three and seven. Interestingly, enzyme content was highest on both polished and passivated AM surfaces, followed by stainless steel and vibratory finish, suggesting that postprocessing and material selection may have influenced early cell differentiation. Nevertheless, ALP content became more homogeneous between groups after the maximum reached at day 14 and after continuously decreasing over the next two weeks, with polishing and vibratory finishing being the only treatments showing statistically higher and lower enzyme expression at days 14 and 21, respectively.

Contrasting with the limited differences in metabolic activity and late ALP production, the amount of calcium deposits resulting from cell differentiation is highly dependent on the material and postprocessing selected (Figure 5f). After the first week of differentiation, all AM postprocessed materials showcased a significantly (*p* < 0.05) higher presence of AR than stainless steel, although calcium deposits rapidly increased over the next week, followed by a more subtle raise throughout all groups at days 21 and 28. During these latter points, it is clear that polishing followed by passivation enhanced the amount of calcium deposits when compared to vibratory finishing. On the other hand, stainless steel displayed a significantly higher AR content at day 14, stagnated 1 week after, and finally rapidly increased on the last timepoint considered. Qualitative XRF imaging (Figure 5g) revealed limited calcium and phosphate deposited over the first week, after which high concentrations of both elements could be found at the center of the samples. Nevertheless, further cultivation led to these centralized deposits becoming more homogeneously distributed over the surface for both elements.

When normalized ALP and AR are compared, it is clear that the rapid increase in enzyme between days 7 and 14 was tied with the quick mineralization at day 14. However, more interesting are the similar trends observed when postprocessing conditions are considered, with the polished and stainless steel exhibiting a higher AR quantification compared with the passivation and stainless-steel samples. ALP is commonly seen as a marker for early mineralization, indicating that rises in enzyme content should be followed by mineral deposition. Generally, this seems to be the case with shifts in ALP leading to similar variations in calcium deposits (Figure 5e), suggesting that cells expressing higher levels of ALP during the first mineralization stages may have mineralized earlier. Nevertheless, ALP is focused on phosphate formation with tissue-nonspecific alkaline phosphatase providing inorganic phosphates through hydrolyzation of pyrophosphate, promoting mineralization.^87^ Thus, differences between ALP and AR during the later stages may be caused by upregulation of other genes involved in mineralization (e.g., osteopontin).^87,88^

**Figure 6:**
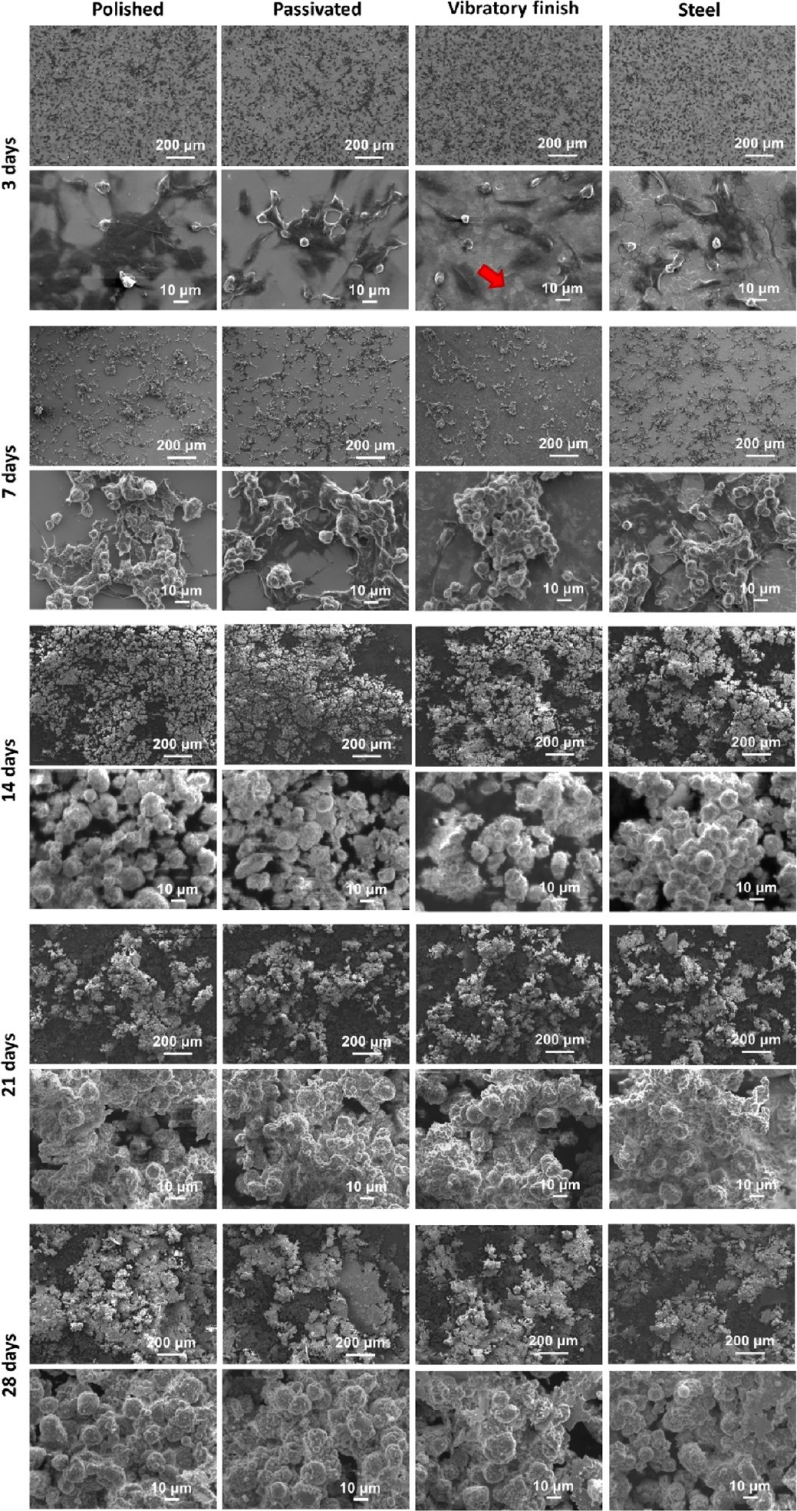
SEM imaging of SAOS-cells deposited on polished, passivated, and vibratory finished additively manufactured titanium surfaces and 316L stainless steel after 3–28 days of cultivation and differentiation.

Comparable degrees of cell confluency can be seen in most of the selected surfaces at day 3 (Figure 6), which also indicated the presence of extracellular matrix in the vibratory finished sample (red arrow in the detailed image). This similar coverage can still be observed after 7 days, although cells seem to start agglomerating under all conditions considered. At day 14, an explosive proliferation of cells and the apparition of large patches that Vieira et al.^89^ had previously confirmed as mineral deposits occur in SAOS-2 cells treated with osteogenic media, which are in agreement with DNA, ALP, and AR. Interestingly, further cultivation led to a slight remodeling of these mineral and cell patches, spreading into smaller agglomerations over the surface in accordance with the XRF mapping. Detailed images of these cells indicate that the visible membranes start developing mineral deposits at day 14; however, these become more prominent at days 21 and 28, with some appearing highly mineralized (Figure 6). This seems to be in agreement with the decrease in ALP after day 14 and rise in AR, which have been previously mentioned as the indicator of calcium nodule formation.^90,91^ It should be noticed that a significant reduction in collagen content is observed (Figure 5d) for all samples displaying prevalent mineralization over time. Previous studies on human osteosarcoma SAOS-2 have shown that these osteosarcoma cells produce a rich extracellular matrix (ECM) composed of collagen, fibronectin, laminin, and proteoglycans with functionality beyond cell growth (e.g., tumorigenesis and metastasis).^92,93^ Although in this study only collagen was measured, this seems to indicate that polishing, passivation, and stainless steel have impaired ECM production while enhancing mineralization. Given that these postprocesses and materials have not significantly affected cell attachment and proliferation, it may be possible to assume that the limited collagen available is enough to support cell growth. Nevertheless, the significantly higher collagen produced by the vibratory finished sample followed by impaired mineral development and reduced ALP expression seems to point out that surface influence in cell behavior is more complex and highly focused on ECM production and differentiation. Thus, further genomic analysis and gene expression tests should be carried out to unravel all pathways driven by AM postprocessing.

Titanium and its alloys are predominantly used in medical applications as a result of their biocompatibility as well as mechanical and chemical stability.^94^ Nevertheless, some clinical applications have showed that integration may not be sufficiently fast, leading to significant research on surface treatments to enhance their natural osseointegration.^57^ Similarly, to bacterial behavior, cell–surface interactions are dependent on the physicochemical properties of the base material and, although correlations can be found in the literature, differences in materials, methods, and contradicting reports complicate the extraction of concrete relationships. For instance, it is generally accepted that optimal bone-to-implant contact and peri-implant bone formation takes place for average *R*_a_ in the 1–1.5 or 3–4 μm ranges.^95−97^ This coupled with in vivo reports of poor integration for smooth machined titanium,^95,96,98^ ∼0.8 μm, and better bone-implant interlocking offered by macrostructured surfaces,^99^ has led to a predominant role of micro and macro roughness in these devices. However, the works of Lavenus et al.,^100^ Li et al.,^32^ and Ting et al.^96^ reveal the ability of cells to interact with submicron and nano-topographic surfaces, *R*_a_ < 0.5 μm, albeit the range of optimal biological response differs between reports. Other studies on smooth surfaces have also shown their ability to enhance spreading and proliferation of osteoblasts^51,101^ which is in agreement with the current study. The results obtained in Figure 4 indicate a limited effect on cell viability and material coverage with surfaces studied revealing a uniform and rapid proliferation. Interestingly, a similar lack of statistical significance in cell viability for different postprocessed titanium samples has already been reported by Bernhardt et al.^51^ In the present study, some statistically significant differences were observed between groups in normalized ALP during the initial stages of cell differentiation and mineralization. In contrast, these become more subdued in the long term, although it is necessary to mention the similarities in ALP between stainless steel and postprocessed AM surfaces. These differ from the rapid increase in mineral deposits showcased by the stainless-steel substrate after 28 days of culturing (Figure 5e,f), similar to the observations of Malheiro et al.^102^ Given that ALP is an indicator of early mineralization and coupled with the large calcium deposits observed,^87^ it seems that cells deposited on this rougher material, *R*_a_ = 95.8 ± 3.8 nm, reached the latter stages of mineralization later than all AM Ti-6Al-4V samples, *R*_a_ = 40–50 nm. Thus, it suggests that mineralization may be influenced if the surface roughness is similar in size to that of bone extracellular matrix, ∼400 to 600 nm, as proposed by the work of by Rafiee et al.^99^ That said, there are a number of differences in chemistry and other physicochemical properties, which may also play a role.

When a biomaterial is submerged in cultured media, interstitial fluids, or blood, protein adsorption takes place before eukaryotic or bacterial cells interact with the substrate.^58,103^ As mammalian cells approach the surface, integrins or transmembrane heterodimeric glycoproteins controlling cell adhesion, shape modifications, proliferation, and migration bind with the resulting layer of proteins.^104^ Thus, it is apparent that protein surface interaction heavily influences cell contact. From Figure 5a, it is clear that protein adsorption has been maximum in both passivated and vibratory finished AM Ti-6Al-4V samples, although the main reason for these differences may be uncertain. It has been previously suggested that an increase in surface roughness and wettability provides a larger surface area of contact for protein adsorption, indicating that topology plays a more fundamental role than chemistry.^35,40,105^ On the other hand, it has been demonstrated that oxidized Ti-Al-V alloys can enhance adsorption of key extracellular matrix proteins.^106^ At the same time, ζ-potential has been suggested to affect protein adsorption and configuration,^107^ with Cai et al.^108^ indicating that lower ζ-potentials may constrain protein adsorption. Interestingly, the results obtained in this manuscript suggest that the surface chemistry may be the main driver behind this interaction. All Ti-6Al-4V samples revealed similar surface finishes, stainless steel being the sample with higher roughness, thus, offering increased area for protein interaction (Figure 1). Nevertheless, no increased protein adsorption could be found on this sample. Consequently, it seems plausible that the passivation and vibratory finishes caused the appearance or larger oxygen-rich species on the surface, which combined with their hydrophobicity may explain the greater protein adsorption. Nevertheless, viability assays did not indicate any significant differences between groups in metabolic activity, suggesting a limited effect of this layer in cell proliferation. This may have resulted from conformational changes in the adsorbed proteins or from strong nonreceptor chemical binding such as hydrogen binding, electrostatic, polar, or ionic interactions, being the main initiator of cell–surface attachment.^31,58^

Studies concerning the effects of both cell attachment and differentiation on some of the aforementioned parameters have been conducted through wettability and surface free energy measurements. Most of the available literature^57,58,109^ has suggested that hydrophilic and, as such, high surface free metals result in high biocompatibility and osteogenic responses, with other in vitro and in vivo studies further supporting these results.^35,110,111^ Part of this effect come from the higher spreadability offered by high wetting surfaces, although the role of surface free energy cannot be neglected. The work of adhesion is dependent on both polar and nonpolar components of the surface free energy; however, it is the γ^AB^ or polar component that mostly affects polar molecules such as water and proteins.^57,112^Figure 5f indicates that as the polar component of the Ti-6Al-4V samples rises and the CA diminishes, the calcium deposits obtained after 14, 21, and 28 days are reduced. Similar trends for postprocessed metallic alloys can be found in the work of Kubies et al.^57^ who subdivided the biological response of different bone-implant materials based on the measured values for the polar component, >8.88, 2.5–7.55, and <2.46 mN/m. The presented results seem to support the previous statements; nevertheless, stainless steel displayed the lowest polar and higher CA measurements, with similar mineralization levels to those of the polished surface at days 14 and 28 (Figure 5e). Consequently, it seems that for the AM Ti-6Al-4V wettability and surface free energy may be a good indicator of cell behavior to guide implant manufacturing, while for the stainless steel, this is driven by a combination of these properties and surface topology.^113^ Although the role of roughness, chemistry, wettability, surface free energy, and surface ζ-potential in biological interactions has been previously recognized in the literature, herein we have shown that this is the main driver for postprocessed titanium AM surfaces which may be used as a tool to develop novel medical devices.

## 4 Conclusions

In the present study, additively manufactured Ti-6Al-4V samples were produced and postprocessed to analyze the fundamental physicochemical properties affecting the biological responses of both eukaryotic and prokaryotic cells. From all variables analyzed (roughness, chemistry, CA, surface free energy, and ζ-potential), variations in *S. epidermidis* colonization were mostly driven by wettability and surface free energy, showcasing a reduced attachment and proliferation on hydrophobic or low acid–base, γ^AB^, substrates. Similarly, mineralization on Ti-6Al-4V AM substrates was directly correlated with the total surface free energy and its acid–base component, albeit optimal bacterial reduction resulted in limited mineralization. These results indicate that surface free energy has a dominant effect on the biological outcome of metallic surfaces; however, it must be recognized that other factors also played an important role while cellular behavior is constricted by the species and time frames selected. The central role of surface free energy could be used to guide the development of future medical devices, although chemical modifications achieved through single postprocessing methods may not be enough to optimize medical devices. As a consequence, it seems plausible that a combination of different physicochemical processes would be required to ensure limited bacterial proliferation while maximizing mammalian cell interactions for optimal biological interactions.
